# Hyphal network whole field imaging allows for accurate estimation of anastomosis rates and branching dynamics of the filamentous fungus *Podospora anserina*

**DOI:** 10.1038/s41598-020-57808-y

**Published:** 2020-02-21

**Authors:** J. Dikec, A. Olivier, C. Bobée, Y. D’Angelo, R. Catellier, P. David, F. Filaine, S. Herbert, Ch. Lalanne, H. Lalucque, L. Monasse, M. Rieu, G. Ruprich-Robert, A. Véber, F. Chapeland-Leclerc, E. Herbert

**Affiliations:** 1 Université de Paris, Laboratoire Interdisciplinaire des Energies de Demain (LIED), UMR 8236 CNRS, F-75013 Paris, France; 2 Université Paris-Saclay, Laboratoire de Mathématiques d’Orsay, CNRS, F-91405 Orsay, France; 3 Université Côte d’Azur, Laboratoire Mathématiques & Interactions J. A. Dieudonné, UMR 7351 CNRS, F-06108 Nice, France; 4 Université Côte d’Azur, Inria, CNRS, LJAD, COFFEE and ATLANTIS teams, F-06902 Valbonne, France; 5 Institut Pasteur, Image Analysis Hub, C2RT, F-75015 Paris, France; 6 CMAP, CNRS, I.P. Paris, F-91128 Palaiseau, France

**Keywords:** Biological techniques, Biophysics

## Abstract

The success of filamentous fungi in colonizing most natural environments can be largely attributed to their ability to form an expanding interconnected network, the mycelium, or thallus, constituted by a collection of hyphal apexes in motion producing hyphae and subject to branching and fusion. In this work, we characterize the hyphal network expansion and the structure of the fungus *Podospora anserina* under controlled culture conditions. To this end, temporal series of pictures of the network dynamics are produced, starting from germinating ascospores and ending when the network reaches a few centimeters width, with a typical image resolution of several micrometers. The completely automated image reconstruction steps allow an easy post-processing and a quantitative analysis of the dynamics. The main features of the evolution of the hyphal network, such as the total length *L* of the mycelium, the number of “nodes” (or crossing points) *N* and the number of apexes *A*, can then be precisely quantified. Beyond these main features, the determination of the distribution of the intra-thallus surfaces (*S*_*i*_) and the statistical analysis of some local measures of *N*, *A* and *L* give new insights on the dynamics of expanding fungal networks. Based on these results, we now aim at developing robust and versatile discrete/continuous mathematical models to further understand the key mechanisms driving the development of the fungus thallus.

## Introduction

Due to their paramount role in most ecosystems and in different sectors of human economy, filamentous fungi have attracted a lot of attention in the last decades. Indeed, these organisms are able to grow in complex environments, leading to their ubiquitous occurrence^1,2^. The success of filamentous fungi in colonizing most natural environments can be largely attributed to their ability to form hyphae, whose width typically ranges from 2 to 20 µm, and which are usually composed of multiple cells separated by septa^3^. At the edge of the fungal colony, apical cells explore new territories in search for food and are mainly involved in nutrient acquisition through the production of extracellular enzymes for the decomposition of organic matter, as well as through the sensing of their local environment^4^. Sub-apical cells generate new hyphae by lateral branching, thus increasing the density and surface area of the colony. In ascomycetes and basidiomycetes, the branches also mediate the hyphal fusion events, or anastomosis, that occur in the thallus and appear to be crucial for the exchange of nutrients and signals between different hyphae within the same colony^4^. This results in an expanding three-dimensional network of interconnected hyphae, known as a mycelium, consisting of hyphal tips (also named “apexes”) in motion, and presenting hyphal branching and fusion^5^. Depending on the species and the environmental conditions, as well as on the nutrient availability and temperature, the mycelial growth rate can typically range from 10 to 100 µm/min. The fungal network operates on spatial scales ranging from few micrometres to many square metres, or even kilometres. In general, they also persist for extended periods of time^6,7^.

It has now been established that the characterization of fungal networks can not be limited to laboratory experiments, as traditional cell and molecular techniques may be expensive, tedious or of limited scope^8^. Mathematical modeling can help and complement the biophysical approaches at the hyphal level, and the use of image analysis have brought major advances in the understanding of the expansion of fungal networks through different combined multidisciplinary approaches. In parallel, branching patterns have been widely studied by both biologists and mathematicians, in order to identify the respective roles of internal and external factors in the induction of the branching process^9,10^. Recently, a lattice-free three-dimensional fungal growth model was proposed, that takes into account the interactions between the *in silico* fungus model and different substrates and media^11^. In these modeling approaches, the mycelium can either be considered as a single entity, defined by its macroscopic emerging features such as the local density of the hyphal network or the total area it covers on a Petri dish^12^; alternatively, it can be seen as a population of individual hyphae and apexes interacting on a microscopic scale. This second point of view allows to study the fine development of a fungal network by following the evolution of a set of specific quantitative parameters, such as the total length of mycelium, the number of apexes, the number of nodes (i.e. the fusion and branching points of the network) and the number of edges (i.e. the hyphal segments). Such approaches have been successfully developed, notably to evaluate the effects of various constraints on the fungal network development, as in the basidiomycete *Phanerochaete velutina*^6^. A system combining image analysis and graph theory^13^ was also developed to monitor the space-time mycelial growth of a variety of fungal species at different image resolutions. More recently, an automated and continuous video microscopy tracking of hyphal growth^14,15^ allowed for a quantitative analysis of the growth rate and morphology of a thallus.

*Podospora anserina* is a coprophilous filamentous ascomycete that grows on herbivore dungs, a highly competitive habitat where several dozens of species are present and feed on partially degraded plant material^16^. *P. anserina* is used as an efficient laboratory model because, (i) it is very easy to grow, (ii) the complete sexual cycle can be obtained *in vitro* in seven days and leads to the production of sexual spores, named ascospores, and (iii) the availability of its genome sequence has enabled the development of several useful tools in molecular and cellular biology, as well as in cytology^16^.

In this work, we deepen our understanding of the growth dynamics of filamentous fungi by analyzing the hyphal network construction of *P. anserina* in a controlled environment thanks to an interdisciplinary approach led by a group of biologists, physicists and applied mathematicians. Temporal series of pictures (each of a few centimers sidelength with a fixed resolution of 1 µm) were produced in order to track the network dynamics starting from the germinating ascospore, which involved an adequate data acquisition system. The image reconstruction and the pre-treatment to digitize and identify the hyphal skeletal network was completely automated to be reproducible and to ease the post-processing to access the most robust characteristics of fungal growth: the total length of the mycelium (*L*), the number of nodes (*N*) and the number of apexes (*A*). The spatio-temporal distribution of branching could then be extracted, as well as original and robust features such as the linear densities of *A* and *N* and the dynamics of the intra-thallus area (*S*_*i*_) distribution. A statistical analysis of the local measures of *N*, *A* and *L* was also performed. Such a systematic spatial and temporal exploration enabled us to estimate a set of key physiological features of the fungal network, including the anatomosis rate and the branching dynamics. Our reproducible experimental set-up to track the thallus growth opens up new avenues for the investigation of the dynamics of expanding fungal networks and may be used to develop feed-backed robust and versatile mathematical models.

## Results

### Apex, node and length growth dynamics

**Figure 1:**
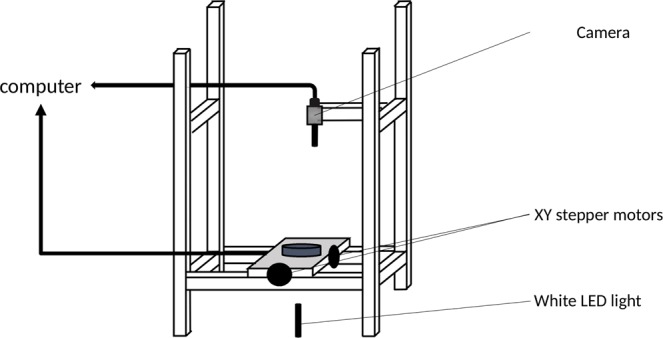
Experimental set-up. The microscopic observations of the fungal growth were made directly on an agar plate. The images were captured with an in-house built microscope, combining a camera (monochromatic 1/3” CMOS sensor with a resolution of 960 × 600 pixels) and a telecentric objective (4× magnification and 65 mm focal length). The resolution of the microscope was measured to be 3.516 µm/pixel. Illumination was achieved using the beam from a white LED light of low intensity passing through the sample.

**Figure 2:**
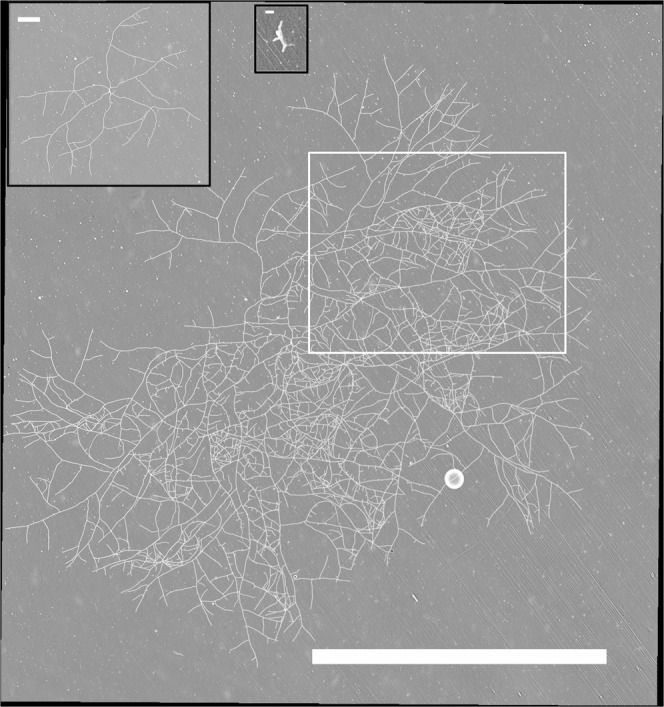
Panoramas of a *P. anserina* fungal network at different development stages. The selected panoramas were taken at *t* = 0 (small black box, germinating ascospore, initial point of experimentation), *t* = 8 h (large black box) and *t* = 18 h (main figure) of the fungal growth from experiment *a*. *t* = 23 h is shown in Fig. 3. The white bars are respectively 100 µm, 1 mm and 10 mm long. The complete collection of panoramas for this experiment is available online in the supplementary material, see^17^. For an enlargement of the white box, see Fig. 4.

**Figure 3:**
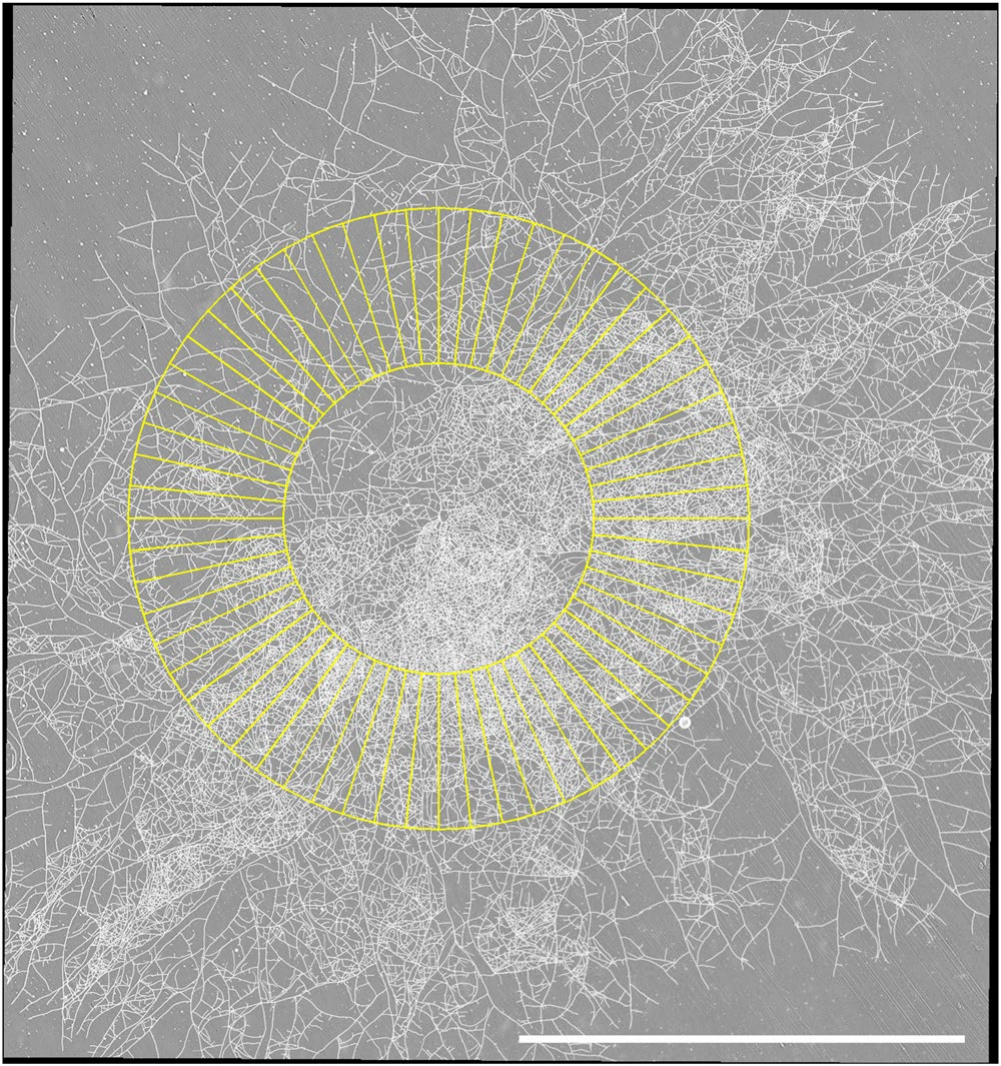
Panorama of the *P. anserina* fungal network at *t* = 23 h from experiment *a*. The white bar is 10 mm long. See earlier acquisitions in Fig. 2. Each panorama corresponds to an assembly of 9 × 12 tiles. The complete collection of panoramas for this experiment is available in the supplementary material, see^17^. In yellow is drawn the intermediate annulus (located at 3516 µm from the center of mass of the thallus) and the corresponding sectors used in the *L*_*l*_ approach, see the text.

**Figure 4:**
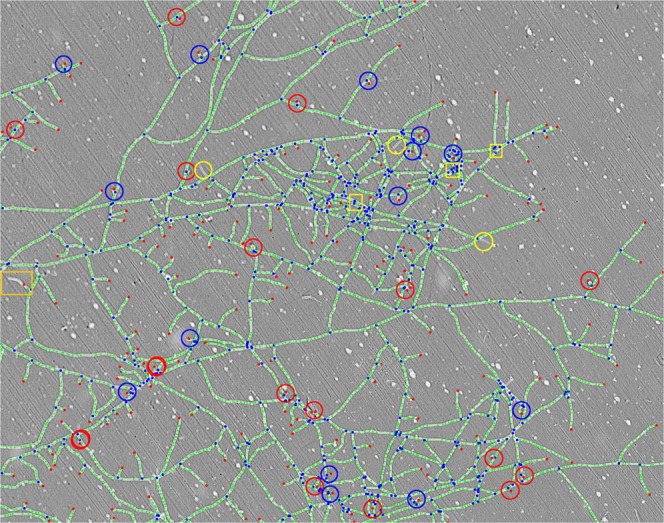
Raw data from experiment *a*, at *t* = 16.3 h corresponding to the white selection in Fig. 2-main), superimposed with the output of the vectorization process. Red dots are apexes and blue dots are nodes connecting three hyphae. The connected paths of green dots constitute the hyphae. Circles are ambiguous apexes *i.e*. it is necessary to observe the growth dynamics in order to decide whether they are true or false. Finally real apexes are shown with blue circles and false positives with red circles. Yellow squares correspond to nodes which do not correspond to biological nodes. Yellow circles correspond to failures to detect a branch, *i.e*. both a branch and a node are missing. The orange box corresponds to undetected hyphal length. The complete collection of comparative images of this selected area corresponding to panoramas 50 to 60 (14.8 to 17.8 hours) can be found online^33^.

As a first step, using the set-up shown in Fig. 1, three series of dynamical panoramas (denoted as *a*, *b* and *c*) were obtained from independently selected germinating ascospores, at 27 °C. A dynamical panorama is a temporal sequence of panoramas ranging from the initial ascospore state until a reference time *T*_*T*_, with lag time τ between two image acquisitions. Experiments were performed on M2 standard culture medium. The experimental set-up allowed us to monitor the development of each *P. anserina* network, starting from the germinating ascospore state and over a period of 21 to 24 hours. Figures 2 and 3 show four particular panoramas, selected from the set of a hundred panoramas obtained in experiment *a*. The complete dynamical development of the fungal network corresponding to this experiment is available online, and the movie can be found in the supplementary material^17^. The full set of panoramas of the three independent experiments were then subject to several image pre-treatment processes in order to digitize and identify the hyphal skeletal network, see Fig. 4.

We fixed the final time *T*_1_ of analysis to be the moment when the first apex reached the edge of the area covered by a panorama. With this convention, the thallus growth was typically observed until it reached *A* ~ 10^3^ apexes, *N*^*g*^ ~ 10^4^ nodes and a total hyphal length *L* of the order of one metre, *i.e*. when the thallus had grown 3 to 4 orders of magnitude larger than its initial ascospore state, see experiments *a*, *b* and *c* in Table 1. Note that due to the ambiguity in interpretation between the overlapping of two hyphae and anastomosis for a fraction of the geometrical nodes *N*_*g*_ obtained by our image processing procedure, we cannot immediately derive the number of biological nodes. In all of that follows, we instead use *N*_*g*_ together with a lower bound *N* on the effective number of biological nodes defined on a subset of the thallus that excludes ambiguous cases. This correction procedure is described in the *Methods*. A proportionality relationship is expected to hold between *N* and *N*^*g*^. We believe this provides a safe estimation of the exponential growth dynamics of nodes for our purposes.

**Table 1:** Summary of the experimental parameters.

Exp.	Symbol	*N*_0_^*g*^	*N*_1_^*g*^	*A*_0_	*A*_1_	*L*_0_	*L*_1_	*S*_1_	*τ*	*T*_1_	*T*_*T*_
		—	—	—	—	[mm]	[mm]	[mm^2^]	[min]	[h]	[h]
*a*	● red	3	4930	5	1399	0.9	1520	117	17.8	18.4	24.0
*b*	* green	3	13976	5	2877	1.0	2847	142	18.0	20.7	21.3
*c*	◆ cyan	3	8898	4	1512	0.8	2519	169	19.5	20.9	23.1

Each of the three experiments, denoted *a* to *c* (the “Exp.” column) were led on M2 medium and are marked with different symbols and colors for ease of reference in the figures (the “Symbol” column). The third and fourth columns display the numbers of geometrical nodes ${N}_{0}^{g}$ = *N*^*g*^(*t* = 0), *N*_1_^*g*^ = *N*^*g*^(*t* = *T*_1_) at time 0 and *T*_1_, and similarly *L*_0_, *L*_1_ and *A*_0_, *A*_1_ are respectively the total length and the number of apexes at initial time *t* = 0 and at time *t* = *T*_1_. *T*_1_ corresponds to the final time of the apexes, nodes and length measurements; our global analysis is thus restricted to *t* < *T*_1_. *T*_*T*_ is the experiment total duration; *τ* denotes the time lag between two image acquisitions; *S*_1_ is the total intra-thallus area at time *T*_1_.

**Figure 5:**
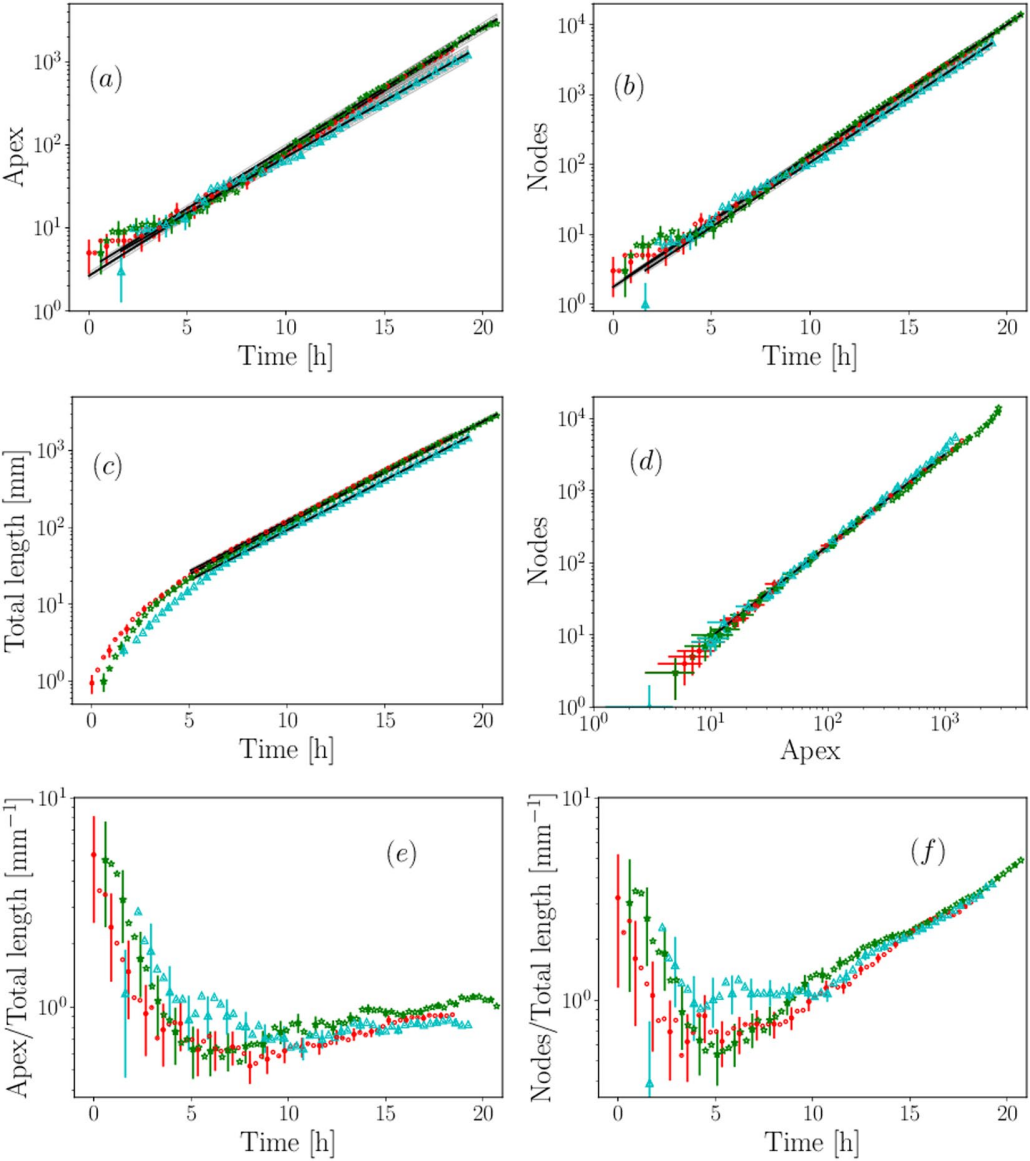
(**a**)-Number of nodes *N*^*g*^, (**b**)-Number of apexes *A* and (**c**)-Total length *L* as a function of time. The ‘final’ time corresponds to the moment when the first apex reaches the edge of the panorama. Experiments *a* (red •), *b* (green *) and *c* (cyan a) are represented, see text and Table 1. Black lines represent the exponential curve fitting in the range 5 < *t* < *T*_1_, see Table 2. The grey shadowing quantifies the uncertainties 2σ. (**d**)-Number of nodes *N*_*g*_ as a function of the number of apexes *A*. The black line is a power law fit in the range 10 < *A* < 10^3^ of the merged experiments *a*, *b* and *c*. The slope was found to be 1.28 ± 0.02. (**e**)-Apexes *A/L* and (**f**)-Nodes *N*^*g*^*/L* densities r*e*lative to the total length as a function of time.

The time evolution of *A*, *L* and *N*^*g*^ is shown in Fig. 5. We see that beyond a typical 5-hour period, these three quantities grow exponentially fast. Our choice of end time *T*_1_ for the analysis does not allow to observe the end of the period of exponential growth (the stationary phase due to the lack of space or resources), that would be marked by a clear slope break in the semi-logarithmic representation. For earliest times (*i.e*., during the lag phase) close to the germination step, a differentiated temporal dependence is observed between the complexity (defined as *A* and *N*^*g*^ counts) and the total mass production (corresponding to the total length *L*). Indeed, *A* and *N*^*g*^ are found to grow exponentially fast almost from the very beginning, and hence the dynamics of the production of complexity appears to be immediately in place. In contrast, the dynamics of length production only reaches an exponential regime after a typical 5-hour lag phase on medium M2. As expected, the slope failure observed in mass production generates a marked minimum on the linear densities of apexes *A*/*L* and nodes *N*^*g*^/*L*, which reach a minimum of 0.5 unit per millimeter after a few hours, see Fig. 5. This minimum marks the end of the initial period of slower growth (during which the elongation of the thallus depends on the initial culture condition) and the start of a global exponential growth regime. From now on and when talking about this regime, the expressions *A* = *A*_0_ exp(*α*_*A*_*t*), *N*^*g*^ = ${N}_{0}^{g}$ exp(${\alpha }_{{N}^{g}}t$) and *L* = *L*_0_ exp(*α*_*L*_*t*) will be used respectively for the quantities *A*, *N*^*g*^ and *L*, where *α*_*i*_ denotes the growth rate of the indexing quantity *i* (see Table 2). Apexes, nodes and length are found to double every 2.11 ± 0.09, 1.61 ± 0.01 and 2.27 ± 0.03 hours, respectively (uncertainties were estimated as twice the standard deviation). In Fig. 5, the number *N*^*g*^ is shown as a function of *A*. The production of complexity (*A* and *N*^*g*^ counts) remains independent of the initial condition over the entire range explored. This suggests that the clock associated to the network increasing complexity is not affected by the same processes as the production of living matter.

**Table 2:** Summary of the growth rate exponents for the apexes *A*, the geometrical nodes *N*^*g*^ and the total length *L* extracted from data shown in Fig. 5.

Exp.	α_A_	${\alpha }_{{N}^{g}}$	α_*L*_
	[h^−1^]	[h^−1^]	[mm.h^−1^]
a	0.341 ± 0.003	0.431 ± 0.002	0.302 ± 0.001
b	0.333 ± 0.002	0.431 ± 0.001	0.305 ± 0.001
c	0.311 ± 0.003	0.425 ± 0.002	0.310 ± 0.001

As the network becomes more complex, to each emerging apex corresponds an emerging node. We should thus expect to have *α*_*N*_ = *α*_*A*_, and of course the associated scaling law *N ∝* ${A}^{{\alpha }_{N}/{\alpha }_{A}}$ would be *α*_*N*_/*α*_*A*_ = 1. However, anastomosis is another effect that *a priori* has to be taken into account. Indeed, when an apex reaches the vicinity of a hypha, these two are expected to merge. This process will appear in the balance as the deletion of one apex and the generation of one node. Over time, the excess of nodes compared to apexes is therefore the signature of anastomosis. When the results of experiments *a*, *b* and *c* are aggregated, the scaling law is found to be ${\alpha }_{{N}^{g}}/{\alpha }_{A}$ = 1.28 ± 0.03, clearly indicating that nodes are produced in excess compared to apexes and hence that anastomosis has a strong impact on the network growth. Since the anastomosis rate is directly related to the rate of production of nodes and apexes, we can write:

1 $${\alpha }_{N}={\alpha }_{b}+{\alpha }_{a}$$

2 $${\alpha }_{A}={\alpha }_{b}-{\alpha }_{a}$$

where the positive constants *α*_*b*_ and *α*_*a*_ are respectively the branching rate and the anastomosis rate. From our estimation of ${\alpha }_{{N}^{g}}$/*α*_*A*_, we obtain *α*_*b*_/*α*_*a*_ = 8.1 ± 0.5. That is, the branching rate is approximately 8 times larger than the anastomosis rate. Let us now to turn to an estimation of the anastomosis and branching rates. As shown in the *Methods*, we can define a subset of nodes *N* in which the processes are only due to biological behaviors. *N* is then proportional to the number of geometrical nodes *N*^*g*^ of this subset and its dynamics will also be exponential.In order to extract the respective values of *α*_*b*_ and *α*_*a*_, we reproduced the fitting procedure on *A* and *N* time series, merging the results of experiments *a*, *b* and *c* with respective pre-factors values *A*_0_ and *N*_0_ for *A(t)* and *N(t)*. Note that the *A(t)* and *N(t)* fittings were constrained to the range *A*_0_ = 3 ± 1 and *N*_0_ = 1.5 ± 1, in order to be consistent with the actual initial conditions. The slopes estimated from the merged experiments are *α*_*A*_ = 0.322 ± 0.002 h^−1^ and *α*_*N*_ = 0.426 ± 0.009 h^−1^, from which we derive an anastomosis rate *α*_*a*_ = 0.05 ± 0.01 h^−1^, together with a branching rate *α*_*b*_ = 0.37 ± 0.01 h^−1^.

The total number of biological nodes of the thallus is comprised in the range 1 − *r* < *N*/*N*^*g*^ < 1 with *r* = 20 ± 5%. The exponentially growing difference between *N*_*g*_ and *A* implies that in the course of a typical thallus growth, the number of nodes reaches the number of apexes (*N*/*A* ∼ 1) after approximately 4.5 to 5 hours, and that the number of nodes becomes dominant (*N*/*A* > 10) after 26 to 29 hours.

### Intra-thallus areas

Working on *closed* intra-thallus space dynamics is an alternative and complementary way to further deepen our understanding of the hyphal network dynamics. In this work, we define an intra-thallus surface *S*_*i*_ as an area completely surrounded by the mycelium (what would be called a *face* in graph theory). The complete thallus is then the sum of these surfaces *S* = Σ*S*_*i*_. The temporal evolution of hyphal growth is characterized by a complexification of its network and thus, by both an increase in its total intra-thallus surface as well as an increase in the number *n* of such regions.

**Figure 6:**
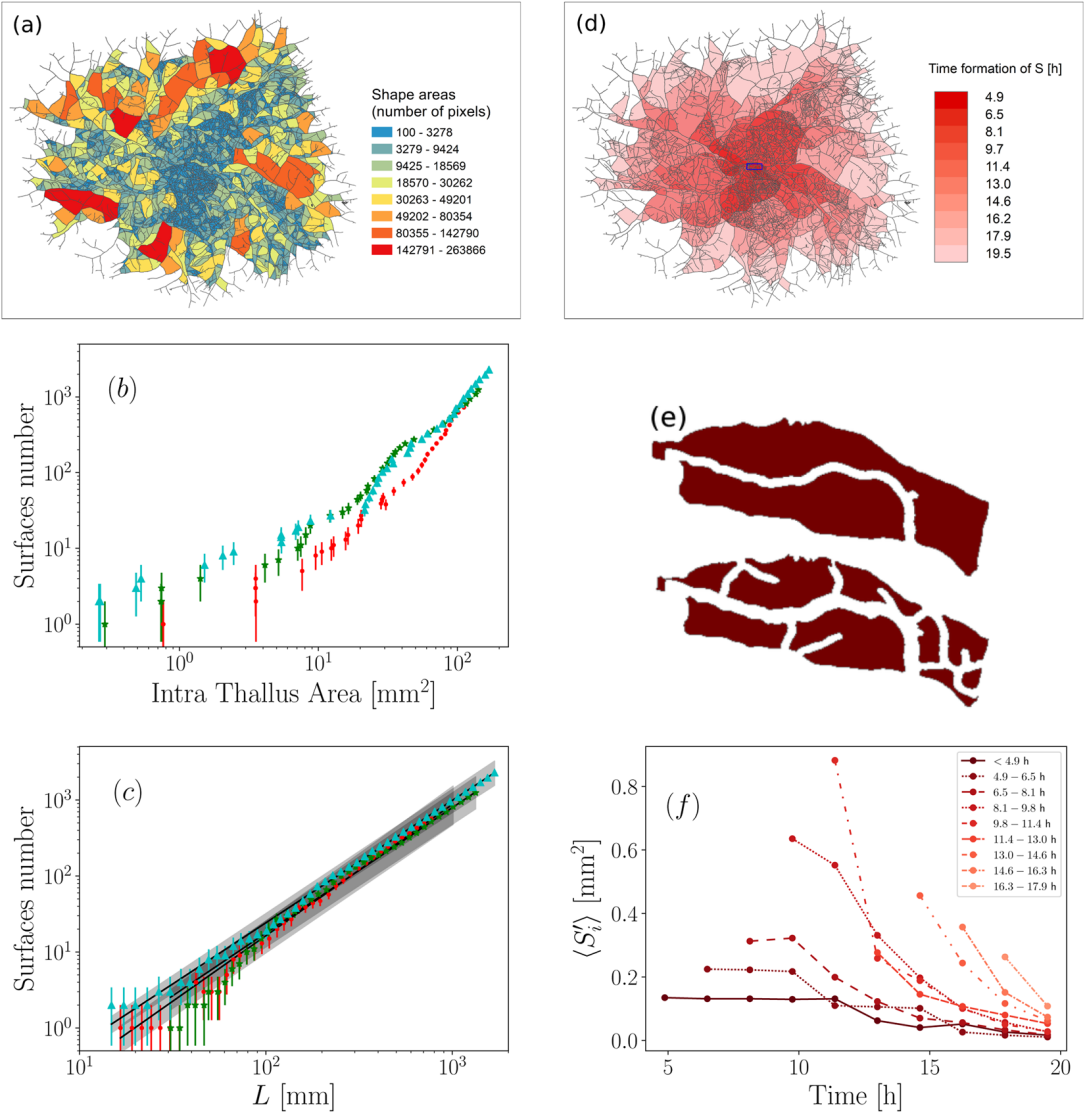
(**a**) Spatial distribution of the intra-thallus areas S_i_ from experiment c. Areas have been discretized into 8 classes, using a natural thresholding method. S corresponds to the entire colored surface (**b**) Evolution of the number of areas as a function of the total intra-thallus areas for experiments a, b and c. (**c)** Evolution of the number of areas as a function of the length of the thallus. Same color code as in Fig. 5. (**d**) Intra-thallus surfaces colored in red shades as a function of their time of appearance: the lightest surfaces are those that are formed the latest (experiment c). (**e**) Spatial distribution of intra-thallus areas S_i_ in the zoomed region represented in blue in (**d**) for t = 4.9 h (top) and t = 17.9 h (bottom). **(f**) Temporal evolution of newly emerged mean intra-thallus mean surfaces (${S}_{i}^{0}$) (experiment c). The surfaces shown with darker colors (towards deep red) are formed earlier when lighter colors are formed later.

Figure 6(a) illustrates the spatial distribution of intra-thallus areas *S*_*i*_, given in pixel counts, for a well developed thallus (namely, experiment *c* at time *T*_1_). In this example, the total intra-thallus surface reaches *S* = 136 mm^2^ and is composed of *n* = 2294 intra-thallus areas, with a mean value of 5.93 × 10^−2^ mm^2^. The values of *S*_*i*_ are highly variable, ranging from 100 pixels to more than 260 × 10^3^ pixel counts, with a relative standard deviation of *σ*_*S*_/*S* ≈ 2.5. As expected, this cartography underlines the presence of a denser network near the center of the thallus compared to the outer border. Moreover, four to five preferential densification directions can also be detected, which are materialized by growth fronts of areas with smaller *S*_*i*_. The evolution of the number *n* of surfaces *S*_*i*_ was extracted from the three series of panoramas, allowing their time evolution to be compared to the total intra-thallus area *S* and to the total length *L*, see Fig. 6(b,c). In particular, it is interesting to observe from Fig. 6(b) that surface counts exhibit a saw-tooth pattern. This highlights the discrepancy between two different processes caused by anastomosis. Indeed, on the one hand, the densification process divides an existing surface into two parts, with the total surface area *S* being preserved. On the other hand, the dynamics of the colonization process only occurs in the outer region of the thallus, and this process consists of adding a new region to the total surface area *S* when an apex reaches another branch to form a new node. The surface *S* is then observed to increase in large steps, largely separated in time, hence the observed saw-tooth pattern. Plotting the number of surfaces *n* as a function of the total length *L* (see Fig. 6(c)), shows a power law behavior of the densification process in the three cases. We report a typical slope of 1.7 ± 0.1 mm^−1^ for the *n*/*L* ratio. This features densification as a regular process, whose dynamics are directly related to length. The spatial subdivision chosen has the double advantage of having a biological and a geometric significance. Indeed the age of formation of the different surfaces increases as we go from the center to the outside of the thallus, as illustrated in Fig. 6(d) which shows the spatial distribution of different formation times of *S*_*i*_, in the range 4.9–19.5 hours. The central region (deep red) is made of an intricate hyphal network whereas a sparser network is observed in the peripheral regions (light red). Moreover, the comparison between Fig. 6(a–d) shows that the preferred densification directions are defined at the very beginning of growth. Let us now turn to the dynamics of surface redrawing. The spatio-temporal dynamics of the fungal network densification can be quantified by observing the dynamics of specific areas, defined as the new surfaces created from an already existing surface. More precisely, we fix a given connected area ${S}_{t}^{{\prime} }$ present in the thallus at some time *t*’ and we follow the fragmentation of this area into smaller surfaces ${S}_{i}^{{\prime} }$ over a given interval of time. To illustrate the procedure, we show in Fig. 6(e) an enlargement of the region shown in the blue rectangle in Fig. 6(d). The initial global surface *S*′ is divided into *n*′ = 2 closed surfaces *S*_*i*_ at *t* = 4.9 h (top). During growth, these two initial surfaces are divided and new surfaces appear (bottom), the average surface being automatically reduced. Between *t* = 4.9 h and *t* = 17.9 h, the number *n*′ of sub-areas grows from 2 to 9, and (${S}_{i}^{{\prime} }$) = ∑ ${S}_{i}^{{\prime} }$/*n*′ decreases from 10905 pix to 1337 pix. This procedure was reproduced over the surface *S* for 9 selected time steps from 4.9 h to 17.9 h, within 1.6 h increments (corresponding to the duration separating five consecutive panoramas). Figure 6(f) shows the temporal evolution of the intra-thallus mean surfaces (${S}_{i}^{{\prime} }$). The lightest colors correspond to more recently formed ${S}_{i}^{{\prime} }$ surfaces. It clearly shows the high spatial and temporal variability of branching dynamics. The first intra-thallus areas formed (darkest plots) are smaller and have a lower branching dynamics than those formed later (between 8.1 and 13.0 hours). Moreover, we distinguish two successive densification phases dominated first by growth and then by densification. Before approximatively 9 hours, exploration of the medium prevails and the branching proccess is reduced. Beyond this time, (${S}_{i}^{{\prime} }$) decreases rapidely and densification appears as the dominating behaviour.

### Statistical tests based on local measures

**Figure 7:**
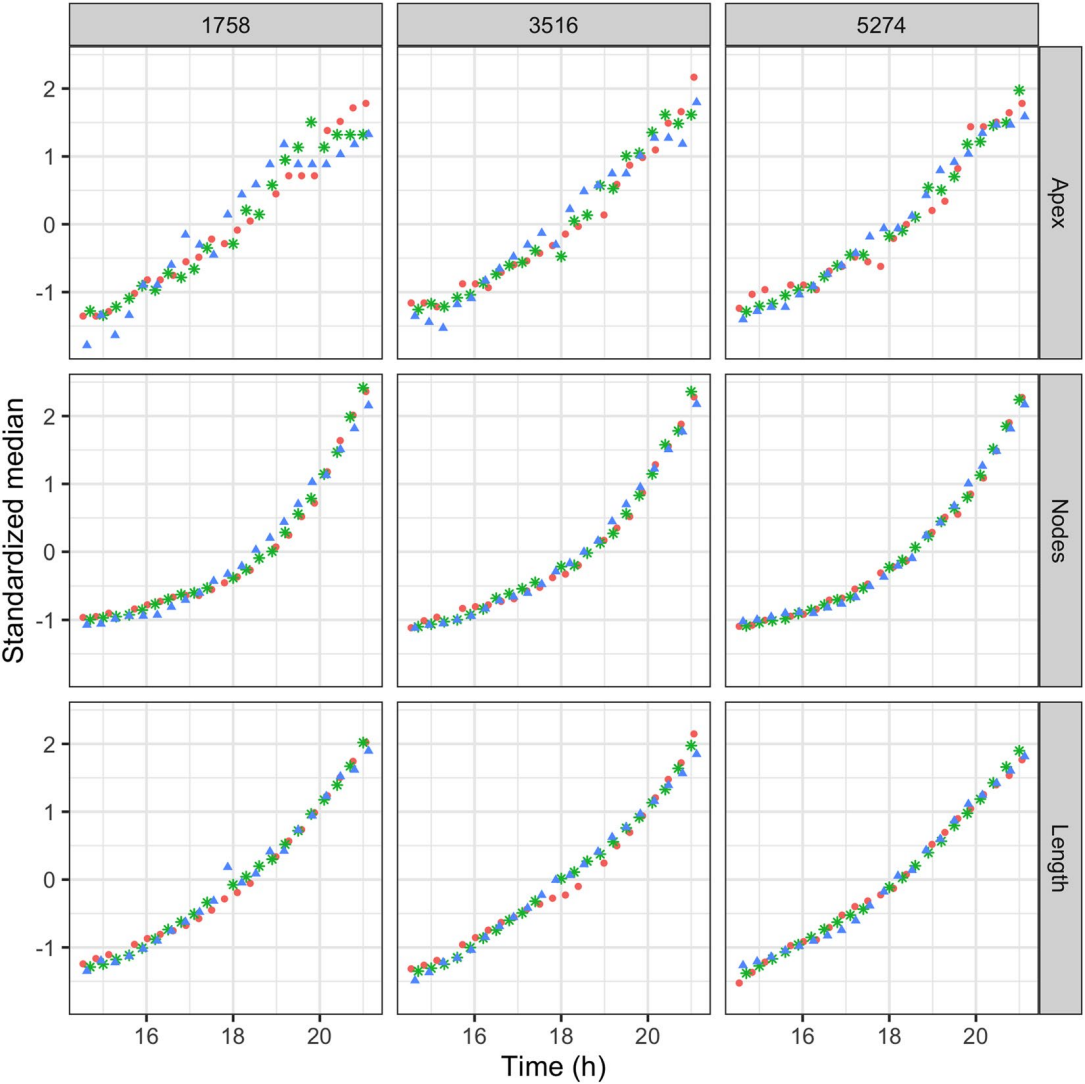
Time series of the median of the standardized distribution of *A*_*i*_*(t)*, *N*_*i*_*(t)* and *L*_*i*_*(t)*, with *i *∈ [0,60] sectors in three distinct annulus of width 3516 µm, see Fig. 3, with an inside diameter of resp. 1758, 3516 and 5274 µm, see the text. Same color code as in Fig. 5.

In order to see whether the local analysis of the network features is consistent with their observed global dynamics, we propose a first quantitative approach to test the homogeneity of the thallus overtime. To this end, we focus on 3 annuli (of 3516 im width), each of them divided into 60 sectors of equal area (see Fig. 3 for an example with a single annulus). These annuli are taken at different distances from the center of mass of the thallus, and therefore the sector areas differ from one annulus to the other. For each experiment, each annulus and each time *t*, we record the number of apexes *A*_*i*_*(t)*, the mycelium length *L*_*i*_*(t)* and the number of nodes *N*_*i*_*(t)* in every sector *i* ∈ {1, …, 60}. We then base our analysis on these local quantities. We observe (not shown) that the global properties (in particular the exponential growth) are also found at a local level, the numerical values of the exponents being naturally dependent on the location and the number of sectors of the annulus. Then, for each experiment, each annulus and each quantity *A*, *L* or *N*, we use the 60 measures obtained at every acquisition time to derive the normalized distributions (*x(t)* − µ_*x*_)/a_*x*_ with *x* respectively *A*_sec_ *(t)*, *L*_sec_ *(t)* or *N*_sec_ *(t)* (the number of apexes, the length and the number of nodes present in a sector), µ_*x*_ the average of *x* and a_*x*_ its standard deviation. In Fig. 7, we show the evolution of the median normalized distributions as a function of time. As we can see, the observed behavior does not seem to depend on the annulus location. The median increases overtime, more or less linearly. The distributions are found to be symmetrical (µ_*x*_ = Median *(x)*) in all cases after approximately 18 hours of growth. As it appears, the filling rate of each sector is not proportional to its initial filling, since the sectors with the highest and the lowest densities grow relatively more slowly than the sectors with intermediate densities. Finally, the sectors with the highest densities converge asymptotically (as time goes on).

**Figure 8:**
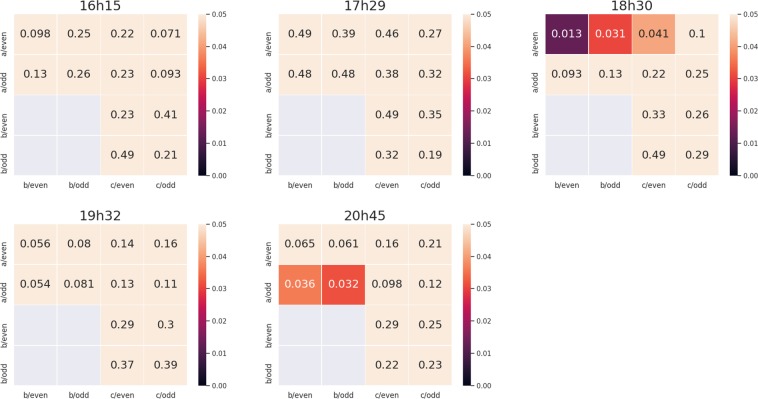
Comparison of experiments *a*, *b* and *c* with local measures – p-values of the Mann-Whitney test (whose null hypothesis is the equality of the distributions) at five different times, see Subsection for more details. The lighter the small box, the higher the p-value.

Our second set of statistical tools aims at testing the reproducibility of our experiments. This time, we use local measures from each of the observed thalli (*a*, *b* and *c*). The results of the comparison of experiments *a*, *b* and *c* are shown on Fig. 8. This comparison is performed using a non-parametric test (*i.e*., no assumption is made on the type of the distributions we are comparing). More precisely, Mann-Whitney tests are performed on the sets

$$({N}_{i}^{(e1)}(t)/{L}_{i}^{(e1)}(t),i\in {I}_{1})\,{\rm{and}}\,({N}_{i}^{(e2)}(t)/{L}_{i}^{(e2)}(t),i\in {I}_{2})$$

where *e*_1_ and *e*_2_ belong to {*a*, *b*, *c*}, *I*_1_ and *I*_2_ may be fixed to {1, 3, …, 59} or to {2, 4, …, 60}, for different times *t*. The conclusion is the following: the null hypothesis, stating that the distributions lying behind the different subsets of data described above are the same, cannot be rejected most of the time. This statistical result validates the reproducibility of our experiments and the reliability of our experimental system.

## Discussion

Our first challenge was to develop a reproducible experimental procedure to track the growth of a fungal network in a controlled environment. This experimental set-up included the control of temperature as well as the monitoring of a constant culture medium, in particular maintaining a sufficient level of humidity, throughout the experiments. A key issue was also the appropriate choice of starting point for the biological sample, between a standardized inoculum of mycelium and a germinating ascospore. Both biological samples may have been used, but germinating ascospores were prefered here because our study was limited to *P. anserina*, and because each selected ascospore was supposed to be in a similar physiological state when germination was initiated. However, ascospores are not necessarily the *ideal* biological starting point if we want to generalize our experimental observations to the development of fungal networks from mutants or from other fungal species unable to make sexual reproduction. In this case, we would rather use a small standardized inoculum, carefully selected so that the physiological state of all the fragments of hyphae should be as comparable as possible. This was successfully conducted by Vidal-Diez de Ulzurrun *et al*.^13^, who compared the network growth of various fungal species by taking an inoculum of few mm cut from the periphery of the mother culture.

A fully automatized acquisition system of multiple images, coupled with an image and graph reconstruction processes was developed. It allowed us to track and analyse the complete mycelium growth from a germinating ascospore to a highly complex network composed of thousands of nodes. The observation system allowed us to record a temporal series of pictures of the network dynamics. Since the sample could be moved along two axes, it gave access to a large area of observation, through reconstituted panoramas of a typical size of 10 mm × 10 mm in this study that may easily be made larger if needed. To summarize, the experimental set-up described here does not require tedious procedures nor expensive equipment, and remains easily upgradable. This is also a versatile system, meaning that it would be possible to study other fungal networks, and certainly some other similar thin biological networks, under various conditions. The observation system was then associated to an image reconstruction and a pre-treatment automated process that allowed us to digitize and identify the hyphal skeletal network, greatly simplified post-processing and then gave an easy access to the different quantities required in the quantitative analysis of the fungal dynamics. This allowed us to study the behavior of the fungal network in a very detailed way, through the measurement of three features: the total length of the mycelium (*L*), the number of nodes (*N*) and the number of apexes (*A*). Our main goal was to extract and compare the evolution in time of these three quantities throughout the global fungal growth on standard culture conditions (M2 medium) from three independent experiments (*a*, *b* and *c*), or equivalently from three different germinating ascospores. From the estimation of the uncertainties associated to *N*, *A* and *L*, as well as from the measurements of *N*, *L* and *A* at each time step, we showed that the experimental process and automated extraction of these quantitative parameters were clearly robust and reproducible. The originality of this study lies in the fact that the observation of the fungal growth was initiated from a state with very few nodes and apexes, individualized from a single ascospore, and not from a multitude of branching points contained in an inoculum. This choice gave access to an accurate characterization of the fungal network dynamics. On standard culture medium, we showed that (i) *A* and *N* exhibited an exponential growth from the beginning of the experiments, whereas it was only observed for *L* after 5 h of experimentation; (ii) the slope discontinuity observed in the mass production led to a marked minimum for the linear densities of apexes (*A/L*) and nodes (*N/L*) and beyond this point, the network growth was no longer affected by the initial culture conditions and became exponential; (iii) the ratio *N/A* increased over time, which is the signature of anastomosis; and (iv) the anastomosis rate (constant through time) could be estimated based on the estimated rates of exponential growth of *A* and *N*.

Moreover, since we demonstrated that our experimental design for the tracking of growth of a fungal network in a controlled environment, associated with the extraction and analysis of quantitative parameters, was clearly robust and reproducible in similar culture conditions (three replicates on M2 medium), it will be of great interest to investigate the fungal growth on various constraints. Preliminary experiments in which growth of the fungal network was followed on a depleted medium (results not shown) seem to show that we are able to extract some interesting features (that need to be confirmed by further experiments) in response to culture starvation, and thus, probably also in response to various constraints or in various environments. As already mentioned, such approaches have been successfully completed, notably by Boddy *et al*.^6^ who explored the adaptive network responses of *P. velutina* to added resources and grazing pressure by collembola through the measurement of the total length of the mycelium, the number of nodes and the number of edges.

We also presented an original approach based on robust tools used in image analysis and geomatics, which allowed us to follow the formation of intra-thallus areas (*S*_*i*_) and their evolution in time. Such an approach is complementary to the global characterization of the fungal network through the analysis of the quantities *A*, *N* and *L* that we described above. It gives access to the spatio-temporal patterning of the network densification and, to a lesser extent, to its complexification. We identified here some interesting features that deserve to be further investigated. In particular, as illustrated in Fig. 6(a–d), it allowed a better understanding of how the fungal network is extended and densified, from the germinating ascospore, on a standard culture medium, assumed to be homogeneous and optimal for the fungal growth. Under these conditions, the fungal network appears denser in the centre of the thallus, and preferential areas of densification are observed from the very beginning of growth. These preferential areas of densification appear to correspond to the first few hyphae produced from the ascospore. It therefore seems that the initial germination conditions durably affect the organization of the thallus. Such a behavior was visible because we chose a germinating ascospore as starting point for the biological sample. We characterized the fungal network at a local scale using biological shapes (or units, *i.e*. the intra-thallus surfaces *S*_*i*_), by selecting some specific areas formed at different time steps identified with their time of appearance. Such an approach presents the advantage of studying the spatio-temporal dynamics of the network densification using a geometric segmentation with a biological significance. Then, the temporal evolution of the intra-thallus mean surfaces (${S}_{i}^{0}$) suggests the existence of different branching strategies and/or different growth rates (namely, the first intra-thallus areas formed are smaller and have a lower branching dynamics than those formed later, see Fig. 6(f)). This result suggests that the expansion process is a priority at the beginning of fungal growth to the detriment of the densification process. These preliminary results need to be further investigated. Note that since the growth of the hyphae is globally centrifugal, the spatio-temporal dynamics of the network densification may also be understood by working on specific ring areas, as we do in the section on local characteristics. Then, for a given area, we should be able to follow its densification and to carefully quantify the length of hyphae growing within this zone. It may also be possible to enlarge the local zone of observation, depending on the experimental needs, for example to compare the network densification between the center of the thallus and the peripheral (and thus more recent) areas. Overall, this approach allowed us to better characterize the spatio-temporal patterning of the network that should result from an efficient compromise between the maximization of the surface occupancy (i.e. densification process) and the increasing production of length. If we replace the fungus in a *in vivo* context, such a behavior fits well with the fact that the fungus is, usually, in competition with other organisms to occupy the colonized area and the use of available resources. This corresponds to a sort of never-ending compromise between the need to occupy the space potentially threatened by other organisms (colonization) and the need to optimally draw resources from where the thallus has developed (densification). Finally, in future work, it will be of high interest to study how the densification and complexification of the thallus pattern is affected when the growth occurs under various constraints. For example, it has been previously reported that, to compensate for nutrient deficiency, the fungal thallus typically first tries to maximize the surface occupancy without relatively increasing its production of length^18^. It is hypothesized that such a behavior allows the filamentous fungi to explore a low-nutrient habitat with rapidly growing, sparsely branched, hyphae and maximize their chances of finding new food sources by maintaining a high extension rate.

We introduced another approach to quantify the fungal network growth, this time at a local scale. This focus on the local network dynamics enabled us to overcome the problems related to our small number of independent repetitions for each experiment by considering instead many small areas, relatively far from each other, in the same thallus. Such local measures were derived within several annuli, fitting the approximate spherical geometry of the thallus. However, such a choice is not suitable for the comparison of media in which the growth speeds of *P. anserina* differ markedly. Improvements can be made in two directions: the duration of the experiments may be extended for depleted media, and the radial (polar) geometry may be replaced by a Cartesian geometry for the local quantification procedures.

Overall, in this study, we went beyond the primary interest of extracting the time-evolving quantities *A*, *N* and *L* to quantify the development of a fungal network. Our analysis enabled us to accurately describe the dynamics and spatial patterning of fungal networks through the quantitative characterization of physiological processes, such as anastomosis and densification.

Such an approach may also have great potential in biotechnological applications exploiting filamentous fungi, such as in the bioremediation of pollutants^19^ or in some fermentation processes^20^. Furthermore, the use of microscopy and image analysis techniques may provide interesting tools to quantify the fungal three-dimensional phenotypes that occur in liquid cultures and may help optimizing the metabolite production, which is influenced by the fungal complex morphology. *P. anserina* is a particularly useful fungus because of its involvement in the decomposition of organic matter, and because it was shown to be able to efficiently degrade lignocellulose, which is a property of crucial importance for the production of biofuels of second generation and renewable chemicals^21–23^. In this context, further experiments to characterize the expanding dynamics of the *P. anserina* fungal network under various and/or limited nutrient resources would be straightforward.

The main limits of this system, as was also reported for other experimental set-ups^13,24^ lie in the facts that (i) the fungal growth is constrained in two dimensions over time, in order to simplify the image analysis and (ii) the substrate is limited to transparent media, *e.g*. agar plates, due to the need for a high contrast between the hyphae and their background. Consequently, the transition to the “fungal real life”, meaning in three dimensions and in a dark and heterogenous microcosm like the soil for saprophytic fungi, is highly challenging. Despite significant progress in image analysis methods, it remains difficult to establish a clear link between *in vitro* measurements and observations of fungal networks in a complex *in vivo* environment.

Nevertheless, the experimental set-up introduced in this paper paves the way for many extensions and generalizations. For example, *P. anserina*, as other fungal species, can express fluorescent proteins that may give access to a three-dimensional imaging of fungal networks in standardized agar media or in opaque structures^16,25^. It may be also coupled with *(i*) a specific tracking of selected hyphae, as previously described^14,26^, (*ii*) the study of local behaviors within the thallus, for instance the comparison between the growth front and the central region of the network, and (*iii*) the global measurement of fungal growth at a macroscale level. Altogether, these observations should give access to a large amount of data allowing the quantitative analysis of fungal growth dynamics and may then be used to develop robust and versatile mathematical models. Such mathematical models are currently under development (A. Véber and R. Catellier, in preparation). They will be used to back the kind of studies presented here with a fine analysis of a somewhat idealized mathematical description of the branching, growth and merging (anastomosis) of hyphae. They will also allow us to link the main parameters characterizing the hyphal evolution at a microscopic level (*e.g*., the hyphal growth rate, the rate of creation of a new exploratory hypha and the *a priori* different rate of densification along the existing network) to more global quantities such as the growth rates of *A* and *N*, or even more macroscopically to the long-term speed of the invasion front formed by the mycelium. Thanks to the current development of a robust statistical methodology associated to this class of models, we will be able to estimate some key parameters of the microscopic evolution that are not directly measurable in experiments and to see how these parameters vary with environmental conditions.

## Methods

### Strains, media, culture conditions and preparation of the biological samples

The *P. anserina* strain used in this study is the ‘S’ wild-type strain^27^. A description of the standard culture conditions, medium composition and the *P. anserina* ascospore production from *in vitro* standard sexual crosses can be accessed online; see http://podospora.i2bc.paris-saclay.fr. Ascospores expelled from mature perithecia were collected on agar covers. For each experiment, a dozen ascospores were individually placed on a germination medium recovered with a cellophane sheet. The germination medium contained ammonium acetate and bacto-peptone that are known to trigger *in vitro* the ascospore germination process^16^. As expected, the germination of ascospores occurred after an incubation time of approximatively 5 hours. The standard synthetic M2 medium contained dextrin (0.5%) as a carbon source. For each experiment, the cellophane sheet containing a selected germinating ascospore was then carefully transferred on an agar medium. In order to limit the loss of medium thickness due to evaporation, and thus the decrease in sharpness of the observations, the agar medium was partially replaced by an equal volume of liquid medium. This area of liquid culture usually reached 50% of the total volume of the agar plate and was located at the edge of the plate, whereas the germinating ascospore was positioned at its center. Then, the biological sample (i.e. the germinating ascospore) was ready to be observed and constituted the initial point of the growing fungal network.

### Observation system

The scheme of the experimental set-up is shown Fig. 1. The optical device consists in a camera located above the sample, equipped with a 960 × 600 pixels CMOS monochromatic sensor and a telecentric lens with a 4x magnification and a depth of field of 10 µm. The pixel-to-microns conversion is 3.516 µm/pix in both directions. The focal length of this lens is 65 mm, which allows the Petri dish to be covered with a standard plate cover, roughly 2–3 mm above the thallus. The covering of the Petri dish minimizes the evaporation of the agar culture medium, which otherwise would generate a decrease in the agar thickness over time and thus a loss of sharpness.

In this work, we limited ourselves to the image acquisition in two spatial dimensions. The growth was vertically constrained to follow the surface of the medium. This was achieved using a rigid sheet of cellophane placed between the culture medium and the germinating ascospore. The fungus can then access the nutrients diffusing through the pores of the cellophane sheet. It is known that *P. anserina* can implement a strategy to cross the cellophane, but this process usually occurs after three days of growth, a period of time well beyond the duration of the observations presented in this work^28^.

Each image (called a tile in what follows) covers a region of 1.09 × 1.74 mm^2^. To access a larger area, it is necessary to move the sensor, as in^13^, or to move the sample. Here, the sample can be moved along 2 axes, thanks to controllable precision micro-control plates, so that the entire surface of a Petri dish may be explored. At the beginning of each experiment, a calibration is operated.

The lighting of the plate is obtained by transmission through the culture medium using a white LED of low intensity (27 mW) and whose lighting region is of a few mm^2^. The diffusion of incident light at the solid/hyphal interface makes the hyphae appear highly contrasted (dark) with respect to the surrounding clearer medium culture, but thicker by a factor of roughly 30% than it actually is. This configuration is desirable for the proper detection of hyphae but generates an ambiguity on the connections, making it hard to distinguish anastomosis from a situation where two hyphae are very close to each other.

In order to improve the efficiency of the reconstruction process, the distance by which the sample is moved between two successive pictures is shorter than the image size, and allows an overlap of 10%. A complete panorama of a surface of 10 × 10 mm^2^ is then typically composed of 9 × 12 tiles.

The travel time between 2 tiles is about 2 seconds and the total time to obtain a panorama is about 10 minutes. In our standard conditions, hyphae grow on average by a few micrometres per minute, and so the fungal network can grow by no more than one hypha width between the beginning and the end of the acquisition. Under standard growth conditions, we found that 17 minutes between the acquisition of two panoramas allowed the trajectory of each apex to be resolved unambiguously. In these experiments, we typically obtained 70 panoramas over a period of 24 hours.

The entire device was placed in the dark in a thermally insulated enclosure. The temperature was regulated at 27 °C thanks to a thermostat connected to copper heat exchangers located inside the enclosure.

### Image processing

#### Reconstruction process

The tiles, independently recorded, were then disposed in rows and columns in order to reconstruct a unique picture — that we call a panorama — of the mycelial network at a given time. We devised (see https://github.com/sebherbert/stitchingMacro) a macro to pre-process (see Figs. 2 and 3) and binarize the images. More precisely, after removing the speckles using a median filter, each time point mosaic was stitched using the *Grid/Collection stitching* method^29^ and subsequently aligned using the *Register Virtual Stack Slices* method^30^.

Binarization was achieved using an automated thresholding method based on the maximum entropy threshold^31^, followed by an opening morphological operation and a particle detection.

The overall image processing procedure then enabled us to produce the time-lapse sequence (*i.e*., the movie) of the growing fungal network with a typical image size of 15 × 15 mm^2^ and a resolution of 3.516 µm/pix. Hence, the whole thallus expanding dynamics could be observed at each time step (of typically 17 min), and the binarized images allowed us to extract all the information about the network structure. We will call them the *growth pictures* in what follows.

#### Vectorization

The successive growth images were then vectorized independently for each time step. For this purpose, we used an adaptation of the library proposed in^32^, based on a triangulation method to determine the centre of mass of each hyphal segment. We obtained a collection of vertices, whose numbers of connections to the network (i.e., their degrees) varied. The degree of a vertex determines its nature. Indeed, an *apex* is only connected once to the network, and hence apexes correspond to vertices of degree 1. Hyphae are composed of a succession of vertices connected twice to the network, and therefore they can be identified with sequences of vertices of degree 2. We call the connection between several hyphae an internal *N*^*g*^, or simply a *N*^*g*^. The growth in number of biological nodes can be achieved through two mechanisms. The first is a branching process due to the appearance of a new apex. The second is a merging process due to the encounter of an apex with a pre-existing hypha (the anastomosis process). In both cases, the corresponding geometrical node detected by the vectorization process will display 3 connections to the network. Consequently, nodes are vertices of degree 3 and we exclude vertices with a higher number of connections to the network. The different steps leading to vectorization were represented in Fig. 4.

For each time step (say, the *t*-th one), we counted the number *A*_*t*_ of apexes and the number ${N}_{t}^{g}$ of geometrical nodes in the current state of the network. This method gives access to the 2-dimensional (2-D) projection of the network. However, the thickness of the network sometimes leads to the overlapping of two crossing hyphae that does not end up in anastomosis. Consequently, it is necessary to distinguish between the number *N*_*g*_ of geometrical nodes identified thanks to the vectorization procedure and the number *N* of biological nodes indeed present in the network. In the next paragraph, we will explain how to evaluate *N* as a function of *N*^*g*^. The local length *L*_*l*_ of the network is defined by the Euclidian distance separating two adjacent vertices. We computed the total length of the network by summing the local lengths $L=\mathop{\sum }\limits_{l=1}^{{n}_{e}}{L}_{l}$ is the index of the edge or line segment, connecting two vertices and *n*_*e*_ is the number of edges. With this method the continuous curvature of a given hypha is represented by a series of line segments, leading to a systematic underestimation of the length of the network. To reduce the error, it is interesting, but time consuming, to increase the number of line segments *n*_*e*_, that is, the number of vertices per unit of length. Using this procedure we approach the real length of the network asymptotically. As a compromize, we chose a setting for which the observed length variation was less than 0.1%. The sum of all these local lengths gives access to the total length *L* of the network.

#### Uncertainties

The uncertainties associated to the counting of the nodes and apexes were estimated independently for each time step. We considered each count *N*_*t*_ and *A*_*t*_ as a Poisson random variable. Recall that the standard deviation of a Poisson random variable with mean λ is $\sigma =\sqrt{\lambda }$. An estimation of the uncertainty associated with the measure of the total length *L* was then obtained by estimating the standard deviation of the distribution of lengths obtained by excluding randomly 10% of the nodes. Reproducing this procedure for different time steps, we then obtained a power law dependence of the estimation of the standard deviation as a function of *L*: σ_*L*_ *∝ L*^0.61^. We used this value, 0.61, as the exponent of the estimator. To determine a more precise standard deviation of the length estimation, a boostraping resampling procedure may be used.

In order to estimate the error in *A* and *N*^*g*^ corresponding to the failure to detect a hypha via the automated image processing, we performed a manual counting of the discrepancy between the raw images (the panoramas) and the result of the vectorization process by superimposing the two representations, as shown in Fig. 4. Because the whole image processing procedure was carried out independently for each panorama (at each acquisition time), we only focused on the 11 panoramas numbered 50 to 60 in experiment *a*, which correspond to the acquisition times ranging from 14.8 to 17.8 hours^33^. More precisely, we selected a particular area in which a direct observation allowed us to accurately count *(i)* the number of apexes *A* and of geometrical nodes *N*^*g*^ detected by the vectorization process and *(ii)* the number of false negatives and false positives for *A* and *N*^*g*^, corresponding to the discrepancy between the raw image and the result of the vectorization process when superimposing the images (Fig. 4). Typically, from the panorama 55, in this selected region, we precisely numbered the detection of 185 apexes and 566 geometrical nodes. Concerning the detection of *A*, each apex surrounded by a circle in Fig. 4 corresponds to a putative false positive (*i.e*. a detected apex whose biological relevance is ambiguous when compared to the raw image). Using previous and subsequent panoramas surrounding panorama 55 that give access to the fungal network dynamics in this area, we were able to determine that for a total of 30 ambiguous apex, only 16 (red circles) were really false positives, generally due to small impurities in contact with a hypha and 14 (blue circles) were in fact true apexes. For the complete sequence of this area from panoramas 50–60 in which ambiguous apexes are marked, please see supplementary data available^33^. These results led to the ratio 16/185 = 0.08. Concerning the detection of *N*^*g*^, each node framed by a yellow square in Fig. 4 corresponds to a putative false positive. We numbered 3 putative false positives in the area on which we focused, that were determined as true false positive after the accurate examination of the surrounding panoramas. Finally, these results led to the ratio 3/566 = 0.005. We also note the detection failure of complete branches, see the 3 yellow circles in Fig. 4. This case corresponds to an out-of-focus situation and both the node and the corresponding apex are not detected when they should be. Moreover, in one case, a small part of the length was not correctly detected in the area surrounded by an orange square. This experimental issue (that does not affect the number of apexes and geometrical nodes) is limited to panorama 55 and is corrected in the following panoramas (see supplementary data^33^). Finally, a similar counting was conducted separately for panoramas 50 and 52. This made it possible to estimate an average ratio of, repectively, 0.09 ± 0.01 and 0.004 ± 0.001 for the wrongly detected apexes and geometrical nodes.

#### Estimation of the number of biological nodes N based on the number of geometrical nodes N^g^

The thallus was pictured in 2 dimensions (2-D), but its growth could not be perfectly constrained in 2-D, which led to a thick network, in which hyphae could cross each other without merging. Consequently, some of the crossings between two hyphae have an ambiguous interpretation. Because the 2-D images that we obtained are projections of this (partially) 3-D dynamics, it is difficult to distinguish between the geometrical connections which are indeed biological, due to branching or anastomosis, and those which are due to the overlapping of two crossing hyphae. A solution that we envision for this problem is to identify the nature of the node in an automated way by following the dynamics of the growth of the corresponding hyphae. *N*^*g*^ is a proxy of *N* in the sense that the two quantities are linked by some relationship. However, in the current state of the method it is not possible to make a perfect segregation between overlaps and mergers (anastomosis). Branching and anastomosis processes are both expected to generate a node with three connexions to the network (degree three). Consequently we deliberately turned down the algorithm’s ability to identify nodes with more than 3 connections. Thus an anastomosis will appear in most cases as an isolated node while an overlap will interpreted by the algorithm as two distinct but very close nodes. In this work, we will limit ourselves to defining a subset in which the nodes are solely due to biology and not to artifacts. An overlap will always be defined by the immediate proximity of two nodes. Thus, by excluding all nodes sufficiently close to each other, we ensure that we only look at the dynamics generated by mergers and branching. We extracted the number ${N}_{n}^{g}$ of nodes within *n* pixels from each other, for various times of the growth of the same thallus (the typical diameter of a hypha structure is 7 pixels). The number of (biological) nodes in the subset described above is then *N* = (1*− r*)*N*^*g*^ with *r* = ${N}_{n}^{g}$/*N*_*g*_. The results obtained for experiment *a* are shown in Table 3.

**Table 3:** Number of nodes ${N}_{n}^{g}$ and fraction *r* = ${N}_{n}^{g}$/*N*^*g*^ within *n* pixels from each other for the same experiment at three times of its growth, for which *N*_*g*_ is respectively equal to 61, 271 and 877.

*n*	5			10			20			50		
*N*_*g*_	61	271	877	61	271	877	61	271	877	61	271	877
${N}_{n}^{g}$	0	0	1	4	9	33	14	54	161	31	116	421
*r*	0	0	0	6	3	4	22	20	18	51	42	48

At constant *n*, ${N}_{n}^{g}$ is found to increase with time, but the ratio *r* = ${N}_{n}^{g}$/*N*_*g*_ is approximatively constant. We can then derive an estimator of the uncertainty σ_*r*_. With *n* = 20 we find *r* = 20% and σ_*r*_ *≈* 1.6%. Keeping a conservative estimate of the uncertainty of 3σ_*r*_, we can then safely conclude that at lest 80% of the nodes are real biological connections with an uncertainty uncertainties Δ*N*_*g*_ = *√N*_*g*_ and Δ*r* = 3σ_*r*_. of approximatively 5%. The uncertainty Δ*N* of *N* can then be derived from the relationship between *N* and *N*_*g*_ and using the

### Reconstruction of the spatial structure of the network

The treatment of the panoramas was performed using automatized GIS tools (Geographic Information System), in which each image is defined as a geographic data. The grey level panoramas are characterized by pixel values of varying intensity. The first processing steps aimed at extracting only the thallus object, and can be summarized as follows: (i) the generation of a binary image using a threshold operation; at this stage, both the thallus and sparse speckles were defined by non-zero pixel values; (ii) the conversion of the input binary image into a polygon feature class: each group of contiguous cells with the same values (0 or 1) was converted into a polygon; all polygons were stored in the same shape-file; (iii) the extraction of the thallus polygons was processed by selecting the polygon object that intersects the center of the mycelium (a predefined object with a point-type geometry that we placed at the center of the thallus). The second processing step was to reconstruct “clean” binary images (rasterization), on which we applied again a vectorization process to convert them into polygon objects. The intra-thallus areas *S* were defined in this study as all zero value polygons that were completely inside the thallus. They were computed (defined here in pixel count) for each panorama, and the following statistics were calculated: sum, mean and number of *S*_*i*_), see Fig. 6.

We defined in this work the surfaces ${S}_{i}^{{\prime} }$ formed at the same time steps (*t −* δ*t*,*t*) as ${S}_{t}^{{\prime} }$ = ∑${S}_{i}^{{\prime} }$, using a duration corresponding to five consecutive panoramas (δ*t* = 1.6 h for experiment *(c)*, see Fig. 6(e,f)). The analyse were started at *t* = 4.9 *h*. In other words, surface ${S}_{{t}_{1}}^{{\prime} }$ with *t*_1_ = 6.5 h was formed between 4.9 and 6.5 h) and is defined as all ${S}_{i}^{{\prime} }$ whose centroids do not intersect the intra-thallus surfaces formed at *t*_0_). The procedure was then reproduced for subsequent time steps.

### Local measures

For each selected panorama, three annuli with the same thickness (3516 µm) were built. Their respective inner *R*_*in*_ and outer *R*_*out*_ radii were set to (*R*_*in*_, *R*_*out*_) = *(*1758,5274*)* µm, *(*3516, 7032*)* and *(*5274, 8790*)*. These annuli were then discretized with equidistant angles, to form a total of 60 angular sectors for each annulus. In order to ensure some independence between the angular sectors, every other sector was kept, leading to two sets of 30 sectors, as shown in Fig. 3. At each time step and for each sector, the number of apexes, the number of nodes and the length of the local network lying inside the sector were computed and denoted respectively by *A*_*i*_*(t)*, *N*_*i*_*(t)* and *L*_*i*_*(t)* for the *i*-th portion at time *t*.

We carried out this procedure for the three panoramas (the three thalli) and we compared the empirical distributions of these local measures for the three thalli. Note that this methodology enables us to explore the long time behavior of the thallus, even when it has become too large to be fully observed.
